# 7-Amino­heptyl­aza­nium iodide

**DOI:** 10.1107/S1600536811037329

**Published:** 2011-09-17

**Authors:** Guido J. Reiss

**Affiliations:** a Institut für Anorganische Chemie und Strukturchemie, Lehrstuhl II: Material- und Strukturforschung, Heinrich-Heine-Universität Düsseldorf, Universitätsstrasse 1, D-40225 Düsseldorf, Germany

## Abstract

The absolute structure of the title compound, [H_3_N-(CH_2_)_7_-NH_2_]I, has been determined from the diffraction experiment, the Flack parameter refining to −0.02 (2). In the crystal, adjacent symmetry-related cations are connected by head-to-tail *R*′H_2_N^+^—H⋯NH_2_ *R* hydrogen bonds, forming chains along [010]. The remaining four H atoms attached to the amino and the aza­nium group form weak hydrogen bonds to neighbouring iodide anions, producing a three-dimensional hydrogen-bonded network. The amino group and the aliphatic chain of the 7-amino­heptyl­aza­nium cation show an exact all-*trans* conformation, within experimental uncertainties. The aza­nium group, to fulfill the needs of hydrogen bonding, is twisted out of the plane defined by the C atoms of the aliphatic chain, the C—C—C—N torsion angle being −65.4 (4)°.

## Related literature

For the crystal structures of α-aza­niumyl-ω-amino­alkanes, see: Luciawati *et al.* (2011 ▶); Pienack *et al.* (2007 ▶); Natarajan *et al.* (1996 ▶); Qian *et al.* (2007 ▶). For α,ω-diaza­niumylalkane-containing compounds, see: Frank & Graf (2004 ▶); Jiang *et al.* (2010 ▶); Reiss (2010 ▶); Reiss & Engel (2002 ▶); Reiss & Engel (2004 ▶); Seidlhofer *et al.* (2010 ▶); Takeoka *et al.* (2005 ▶); Vizi *et al.* (2006 ▶). For dye-sensitized solar cells, see: Yang *et al.* (2011 ▶); Gorlov & Kloo (2008 ▶); Grätzel (2004 ▶). For graph-set analysis, see: Etter *et al.* (1990 ▶). For the profile fit on the powder diffraction data, see: Kraus & Nolze (2000 ▶). For background to hydrogen bonds, see: Steiner (2002 ▶).
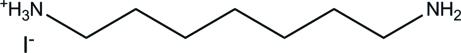


## Experimental

### 

#### Crystal data

C_7_H_19_N_2_^+^·I^−^
*M* *_r_* = 258.14
Monoclinic, 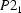

*a* = 5.53418 (8) Å
*b* = 18.7308 (3) Å
*c* = 5.51570 (8) Å
β = 95.2195 (14)°
*V* = 569.39 (2) Å^3^
*Z* = 2
Mo *K*α radiation
μ = 2.76 mm^−1^
*T* = 290 K
0.77 × 0.41 × 0.24 mm

#### Data collection

Oxford Diffraction Xcalibur Eos diffractometer
Absorption correction: analytical [*CrysAlis PRO* (Oxford Diffraction, 2009 ▶); analytical numeric absorption correction using a multifaceted crystal model based on expressions derived by Clark & Reid (1995 ▶)] *T*_min_ = 0.227, *T*_max_ = 0.543
31566 measured reflections
2331 independent reflections
2325 reflections with *I* > 2σ(*I*)
*R*_int_ = 0.028

#### Refinement

*R*[*F* ^2^ > 2σ(*F* ^2^)] = 0.017
*wR*(*F* ^2^) = 0.039
*S* = 1.03
2331 reflections
112 parameters
6 restraints
H atoms treated by a mixture of independent and constrained refinement
Δρ_max_ = 0.33 e Å^−3^
Δρ_min_ = −0.48 e Å^−3^
Absolute structure: Flack (1983 ▶), 1130 Friedel pairs
Flack parameter: −0.02 (2)

### 

Data collection: *CrysAlis PRO* (Oxford Diffraction, 2009 ▶); cell refinement: *CrysAlis PRO*; data reduction: *CrysAlis PRO*; program(s) used to solve structure: *SHELXS97* (Sheldrick, 2008 ▶); program(s) used to refine structure: *SHELXL97* (Sheldrick, 2008 ▶); molecular graphics: *DIAMOND* (Brandenburg, 2010 ▶); software used to prepare material for publication: *publCIF* (Westrip, 2010 ▶).

## Supplementary Material

Crystal structure: contains datablock(s) I, global. DOI: 10.1107/S1600536811037329/wn2452sup1.cif

Structure factors: contains datablock(s) I. DOI: 10.1107/S1600536811037329/wn2452Isup2.hkl

Supplementary material file. DOI: 10.1107/S1600536811037329/wn2452Isup3.mol

Supplementary material file. DOI: 10.1107/S1600536811037329/wn2452Isup4.cml

Additional supplementary materials:  crystallographic information; 3D view; checkCIF report

## Figures and Tables

**Table 1:** Hydrogen-bond geometry (Å, °)

*D*—H⋯*A*	*D*—H	H⋯*A*	*D*⋯*A*	*D*—H⋯*A*
N1—H11⋯I1	0.88 (2)	2.98 (4)	3.738 (4)	145 (5)
N1—H12⋯I1^i^	0.90 (2)	2.88 (3)	3.706 (3)	153 (3)
N2—H21⋯N1^ii^	0.90 (2)	1.87 (3)	2.740 (4)	164 (5)
N2—H22⋯I1^iii^	0.87 (2)	2.72 (2)	3.579 (3)	170 (3)
N2—H23⋯I1^iv^	0.90 (2)	2.83 (2)	3.646 (3)	152 (2)

Symmetry codes: (i) 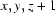; (ii) 
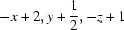
; (iii) 
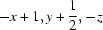
; (iv) 
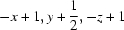
.

**Fig. 1.**
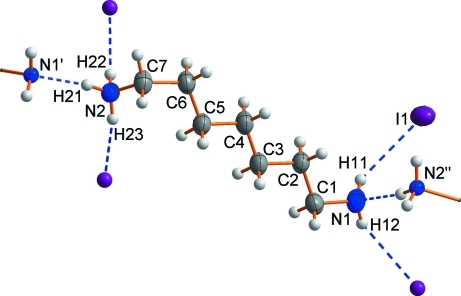
The structure of the asymmetric unit, showing 50% probability displacement ellipsoids. Hydrogen atoms are shown as spheres of arbitrary radius. Symmetry-related neighbouring atoms are drawn with arbitrary radius and dashed lines indicate hydrogen bonds. Symmetry codes : ' = 2 - x, 1/2 + y, 1 - z, '' = 2 - x, 1/2 + y, 1 - z.

**Fig. 2.**
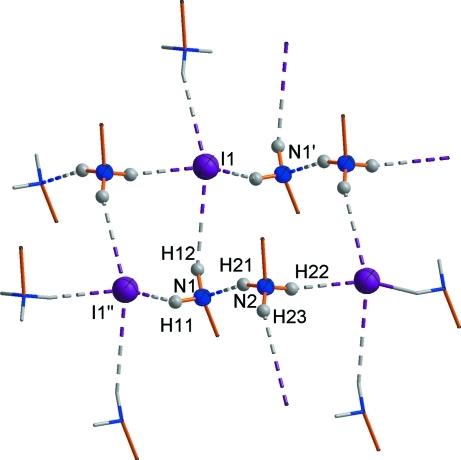
Hydrogen bonding ring motifs. Graph-sets: R24(8) and R46(12)) of the hydrophilic part of the structure are shown. Symmetry codes: ' = 1 - x, 1/2 + y, -z, '' = 1 - x, 1/2 + y, 1 - z.

**Fig. 3.**
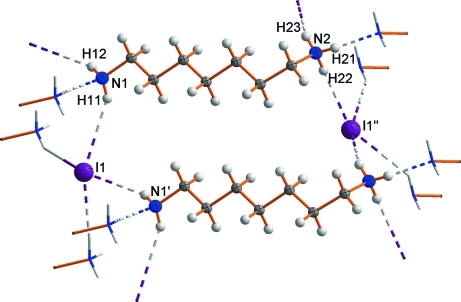
Hydrogen bonding motif of neighboring 7-aminoheptylazanium connected by iodide anions, graph set R24(24). Symmetry codes: ' = 1 - x, 1/2 + y, 2 - z, '' = x, 1 + y, 1 + z.

**Fig. 4.**
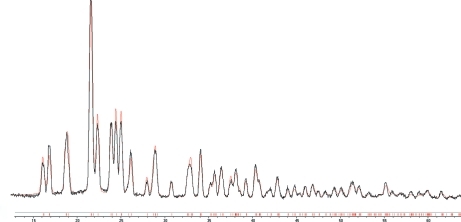
Powder diffraction diagram of the title compound (black line: experimental; red line: profile fit).

## supplementary crystallographic information

### Comment

There is general interest in diazanium iodides because it is well documented that they have a significant influence on the I_3_^-^/I^-^ redox system in binary ionic liquids, which are used as electrolytes for dye-sensitized solar cells (Yang *et al.*, 2011; Gorlov & Kloo, 2008; Grätzel, 2004). Most structures reported in the α,ω-diaminoalkane/HX system are composed of α,ω-diazaniumylalkane dications and complex counteranions. Salts of α,ω-diazaniumylalkanes represent an interesting class of organic-inorganic hybride materials, with a number of different structure design examples: hydrogen-bonded frameworks as host systems for unusual species (Frank & Graf); layered materials (Takeoka *et al.*, 2005), large-pore zeolites (Jiang *et al.*, 2010); non-metal frameworks (*e.g.* Vizi *et al.*, 2006) and metal-frameworks (Seidlhofer *et al.*, 2010).

Our longstanding interest in the structural chemistry of α,ω-diazaniumylalkanes is focused on their versatility as templates for the synthesis of new polyiodides (Reiss & Engel, 2002; Reiss & Engel, 2004; Reiss, 2010). However, only a limited number of high-quality crystal structure determinations on α-azaniumyl-ω-aminoalkane salts have been described (Luciawati *et al.*, 2011; Pienack *et al.*, 2007). Furthermore, the positions of the hydrogen atoms of the hydrogen bond donating groups are not well resolved in all cases (Natarajan *et al.*, 1996, Qian *et al.*, 2007).

This contribution presents a rare example of a crystal structure of an α-azaniumyl-ω-aminoalkane without any disorder. The asymmetric unit of the title compound consists of one 7-aminoheptylazanium cation and one iodide anion. The bond lengths and angles within the organic cation are, with C—C bond lengths between 1.497 (5) Å to 1.517 (4) Å and slightly shorter C—N distances, 1.462 (4) Å and 1.481 (4) Å, as expected. The azanium group, to fulfill the needs of hydrogen bonding, is twisted out of the plane defined by the carbon atoms of the *all-trans* conformation aliphatic chain, the C5—C6—C7—N2 torsion angle being -65.4  (4)° (Fig.1 and Fig. 3)

Cations are connected to symmetry-related units by head-to-tail *R*'H_2_N^+^—H···NH_2_R hydrogen bonds. As a result of this primary connection, one-dimensional *zigzag* chains along [010] are formed (Fig. 1). According to a generally accepted classification (Steiner, 2002), these N^+^—H···N hydrogen bonds can be described as medium strong. Both hydrogen atoms of the amino group and two of the three hydrogen atoms of the azaniumyl group form hydrogen bonds with neighbouring iodide anions. These weak N—H···I hydrogen bonds (Table 1) connect the above-mentioned chains into a three-dimensional framework (Fig. 2 and 3). This framework can be classified by graph sets (Etter *et al.* 1990) as built of two smaller ring motifs [*R*^2^_4_(8) and *R*^4^_6_(12); (Fig. 2)] in the hydrophilic region of the structure and a ring motif *R*^2^_4_(24) that includes the alkyl chains (Fig. 3).

### Experimental

7-Aminoheptylazanium iodide, (H_3_N-(CH_2_)_7_-NH_2_)I was prepared by dissolving 1.77 mmol (0.23 mL) 1,7-diaminoheptane in 1 ml concentrated (57%) hydroiodic acid at room temperature. From this solution crystalline raw material was obtained by evaporation within a few days at room temperature. Recrystallization from fresh hydroiodic acid (57%) yielded block-shaped, almost colourless crystals.

Depending on the reaction conditions, the title compound is sometimes contaminated with a small amount of the dark-coloured α,ω-diazaniumylheptane tetraiodide, (H_3_N-(CH_2_)_7_-NH_3_)I_4_ (Reiss, 2010). To verify the purity of the synthesized material, powder diffraction data of a representive part of the bulk phase were collected on a *Huber G600* diffractometer (transmission, Cu *K*α1, step width: 0.03°, 20 sec./step). A profile fit (Kraus & Nolze, 2000) on the powder diffraction data based on the structure model obtained from the single-crystal experiment proved the identity of the bulk phase with the investigated single-crystal (Fig. 4). This finding is supported by the Raman spectrum collected which does not show the I_4_^2-^-specific absorption band at 175 cm^-1^.

### Refinement

All hydrogen atoms were located from a difference Fourier synthesis. The positional parameters of hydrogen atoms of the NH_2_ and the NH_3_ group were refined with soft N—H distance restraints; the final range of N—H distances is 0.87 (2) - 0.90 (2) Å. All  hydrogen atoms of the CH_2_ groups were refined using a riding model; C—H = 0.97 Å and *U*_iso_(H) = 1.2*U*_eq_(C). Anisotropic displacement parameters of all non-hydrogen atoms and individual isotropic displacement parameters for all hydrogen atoms involved in the hydrogen bonds were refined unrestrictedly. The Flack parameter refined to -0.02 (2).

### Crystal data

**Table:**

C_7_H_19_N_2_^+^·I^−^	*F*(000) = 256
*M**_r_* = 258.14	*D*_x_ = 1.506 Mg m^−^^3^
Monoclinic, *P*2_1_	Mo *K*α radiation, λ = 0.71073 Å
Hall symbol: P 2yb	Cell parameters from 29947 reflections
*a* = 5.53418 (8) Å	θ = 3.3–32.6°
*b* = 18.7308 (3) Å	µ = 2.76 mm^−^^1^
*c* = 5.51570 (8) Å	*T* = 290 K
β = 95.2195 (14)°	Block, colourless
*V* = 569.39 (2) Å^3^	0.77 × 0.41 × 0.24 mm
*Z* = 2	

### Data collection

**Table:**

Oxford Diffraction Xcalibur Eos diffractometer	2331 independent reflections
Radiation source: fine-focus sealed tube	2325 reflections with *I* > 2σ(*I*)
Equatorial mounted graphite monochromator	*R*_int_ = 0.028
Detector resolution: 16.2711 pixels mm^-1^	θ_max_ = 26.5°, θ_min_ = 4.9°
ω scans	*h* = −6→6
Absorption correction: analytical [*CrysAlis PRO* (Oxford Diffraction, 2009); analytical numeric absorption correction using a multifaceted crystal model based on expressions derived by Clark & Reid (1995)]	*k* = −23→23
*T*_min_ = 0.227, *T*_max_ = 0.543	*l* = −6→6
31566 measured reflections	

### Refinement

**Table:**

Refinement on *F*^2^	Hydrogen site location: inferred from neighbouring sites
Least-squares matrix: full	H atoms treated by a mixture of independent and constrained refinement
*R*[*F*^2^ > 2σ(*F*^2^)] = 0.017	*w* = 1/[σ^2^(*F*_o_^2^) + (0.010*P*)^2^ + 0.450*P*] where *P* = (*F*_o_^2^ + 2*F*_c_^2^)/3
*wR*(*F*^2^) = 0.039	(Δ/σ)_max_ = 0.001
*S* = 1.03	Δρ_max_ = 0.33 e Å^−^^3^
2331 reflections	Δρ_min_ = −0.48 e Å^−^^3^
112 parameters	Extinction correction: *SHELXL97* (Sheldrick, 2008), Fc^*^=kFc[1+0.001xFc^2^λ^3^/sin(2θ)]^-1/4^
6 restraints	Extinction coefficient: 0.0965 (15)
Primary atom site location: structure-invariant direct methods	Absolute structure: Flack (1983), 1130 Friedel pairs
Secondary atom site location: difference Fourier map	Flack parameter: −0.02 (2)

### Special details

**Table:**

Experimental. The Raman spectrum was measured using a *Bruker MULTIRAM* spectrometer (Nd:YAG-Laser at 1064 nm; RT-InGaAs-detector); 4000–70 cm^-1^: 3326(w), 3259(w), 2958(m), 2896(s), 2882(s), 2850(s), 2761(w), 1590(w), 1542(w), 1479(m), 1466(m), 1445(s), 1347(w), 1304(m), 1067(m), 1039(m), 961(w), 913(w), 858(w), 838(w), 340(w), 286(w), 253(w), 109(s). IR spectroscopic data were collected on a *Digilab FT3400* spectrometer using a MIRacle ATR unit (Pike Technologies); 4000–560 cm^-1^: 3321(m), 3258(m), 3021(m, br), 2923(s), 2853(s), 1645(m, sh), 1568(m, br), 1487(m), 1465(m), 1384(m), 1359(w), 1334(m, sh), 1302(m), 1244(w), 1156(w), 929(w, br), 817(w), 723(w).
Geometry. All e.s.d.'s (except the e.s.d. in the dihedral angle between two l.s. planes) are estimated using the full covariance matrix. The cell e.s.d.'s are taken into account individually in the estimation of e.s.d.'s in distances, angles and torsion angles; correlations between e.s.d.'s in cell parameters are only used when they are defined by crystal symmetry. An approximate (isotropic) treatment of cell e.s.d.'s is used for estimating e.s.d.'s involving l.s. planes.
Refinement. Refinement of *F*^2^ against ALL reflections. The weighted *R*-factor *wR* and goodness of fit *S* are based on *F*^2^, conventional *R*-factors *R* are based on *F*, with *F* set to zero for negative *F*^2^. The threshold expression of *F*^2^ > σ(*F*^2^) is used only for calculating *R*-factors(gt) *etc*. and is not relevant to the choice of reflections for refinement. *R*-factors based on *F*^2^ are statistically about twice as large as those based on *F*, and *R*- factors based on ALL data will be even larger.

### Fractional atomic coordinates and isotropic or equivalent isotropic displacement parameters (Å^2^)

**Table:**

	*x*	*y*	*z*	*U*_iso_*/*U*_eq_	
I1	0.09785 (3)	0.252871 (19)	0.24986 (3)	0.06035 (9)	
N1	0.4322 (6)	0.34300 (16)	0.7843 (6)	0.0548 (7)	
H11	0.330 (8)	0.342 (3)	0.653 (6)	0.108 (19)*	
H12	0.335 (7)	0.337 (2)	0.905 (6)	0.075 (12)*	
C1	0.5909 (7)	0.40516 (19)	0.8220 (7)	0.0524 (8)	
H1A	0.7136	0.3956	0.9550	0.063*	
H1B	0.4962	0.4458	0.8673	0.063*	
C2	0.7123 (7)	0.42306 (16)	0.5974 (7)	0.0486 (7)	
H2A	0.5885	0.4340	0.4667	0.058*	
H2B	0.8003	0.3814	0.5490	0.058*	
C3	0.8869 (7)	0.48553 (17)	0.6285 (7)	0.0515 (8)	
H3A	0.7997	0.5269	0.6806	0.062*	
H3B	1.0128	0.4742	0.7567	0.062*	
C4	1.0042 (7)	0.50457 (18)	0.4032 (7)	0.0527 (8)	
H4A	0.8785	0.5151	0.2739	0.063*	
H4B	1.0945	0.4636	0.3528	0.063*	
C5	1.1744 (6)	0.56805 (17)	0.4352 (7)	0.0488 (7)	
H5A	1.0862	0.6083	0.4939	0.059*	
H5B	1.3049	0.5565	0.5586	0.059*	
C6	1.2828 (7)	0.58973 (17)	0.2052 (7)	0.0533 (8)	
H6A	1.1515	0.5989	0.0803	0.064*	
H6B	1.3756	0.5498	0.1506	0.064*	
C7	1.4460 (7)	0.65486 (18)	0.2270 (7)	0.0517 (8)	
H7A	1.5742	0.6471	0.3563	0.062*	
H7B	1.5210	0.6612	0.0763	0.062*	
N2	1.3108 (5)	0.72044 (13)	0.2795 (5)	0.0463 (6)	
H21	1.416 (6)	0.755 (2)	0.249 (6)	0.087 (12)*	
H22	1.196 (5)	0.7290 (18)	0.164 (5)	0.066 (12)*	
H23	1.253 (5)	0.7189 (16)	0.427 (4)	0.047 (8)*	

### Atomic displacement parameters (Å^2^)

**Table:**

	*U*^11^	*U*^22^	*U*^33^	*U*^12^	*U*^13^	*U*^23^
I1	0.06054 (12)	0.07533 (14)	0.04638 (11)	−0.01293 (14)	0.01147 (7)	0.00061 (17)
N1	0.0556 (17)	0.0441 (14)	0.067 (2)	−0.0089 (14)	0.0171 (17)	0.0002 (13)
C1	0.061 (2)	0.0411 (17)	0.0565 (19)	−0.0083 (15)	0.0117 (17)	−0.0083 (14)
C2	0.0564 (19)	0.0362 (15)	0.055 (2)	−0.0084 (13)	0.0130 (16)	−0.0013 (13)
C3	0.057 (2)	0.0381 (16)	0.060 (2)	−0.0103 (14)	0.0082 (17)	−0.0036 (14)
C4	0.0567 (19)	0.0385 (15)	0.064 (2)	−0.0104 (14)	0.0097 (19)	−0.0037 (16)
C5	0.0537 (19)	0.0380 (16)	0.0556 (19)	−0.0085 (13)	0.0102 (16)	−0.0005 (13)
C6	0.062 (2)	0.0372 (16)	0.063 (2)	−0.0075 (15)	0.0138 (18)	−0.0065 (14)
C7	0.0499 (18)	0.0391 (16)	0.068 (2)	−0.0047 (14)	0.0155 (17)	−0.0021 (14)
N2	0.0582 (16)	0.0370 (11)	0.0448 (14)	0.0007 (12)	0.0106 (12)	0.0018 (10)

### Geometric parameters (Å, °)

**Table:**

N1—C1	1.462 (4)	C4—H4B	0.9700
N1—H11	0.877 (19)	C5—C6	1.508 (5)
N1—H12	0.898 (19)	C5—H5A	0.9700
C1—C2	1.500 (5)	C5—H5B	0.9700
C1—H1A	0.9700	C6—C7	1.516 (5)
C1—H1B	0.9700	C6—H6A	0.9700
C2—C3	1.517 (4)	C6—H6B	0.9700
C2—H2A	0.9700	C7—N2	1.481 (4)
C2—H2B	0.9700	C7—H7A	0.9700
C3—C4	1.496 (5)	C7—H7B	0.9700
C3—H3A	0.9700	N2—H21	0.90 (2)
C3—H3B	0.9700	N2—H22	0.871 (18)
C4—C5	1.517 (4)	N2—H23	0.901 (18)
C4—H4A	0.9700		
			
C1—N1—H11	118 (4)	H4A—C4—H4B	107.7
C1—N1—H12	112 (3)	C6—C5—C4	113.8 (3)
H11—N1—H12	103 (4)	C6—C5—H5A	108.8
N1—C1—C2	111.6 (3)	C4—C5—H5A	108.8
N1—C1—H1A	109.3	C6—C5—H5B	108.8
C2—C1—H1A	109.3	C4—C5—H5B	108.8
N1—C1—H1B	109.3	H5A—C5—H5B	107.7
C2—C1—H1B	109.3	C5—C6—C7	115.4 (3)
H1A—C1—H1B	108.0	C5—C6—H6A	108.4
C1—C2—C3	114.1 (3)	C7—C6—H6A	108.4
C1—C2—H2A	108.7	C5—C6—H6B	108.4
C3—C2—H2A	108.7	C7—C6—H6B	108.4
C1—C2—H2B	108.7	H6A—C6—H6B	107.5
C3—C2—H2B	108.7	N2—C7—C6	112.0 (3)
H2A—C2—H2B	107.6	N2—C7—H7A	109.2
C4—C3—C2	114.2 (3)	C6—C7—H7A	109.2
C4—C3—H3A	108.7	N2—C7—H7B	109.2
C2—C3—H3A	108.7	C6—C7—H7B	109.2
C4—C3—H3B	108.7	H7A—C7—H7B	107.9
C2—C3—H3B	108.7	C7—N2—H21	102 (3)
H3A—C3—H3B	107.6	C7—N2—H22	111 (2)
C3—C4—C5	113.7 (3)	H21—N2—H22	100 (3)
C3—C4—H4A	108.8	C7—N2—H23	112.1 (19)
C5—C4—H4A	108.8	H21—N2—H23	119 (3)
C3—C4—H4B	108.8	H22—N2—H23	112 (3)
C5—C4—H4B	108.8		
			
N1—C1—C2—C3	−177.8 (3)	C3—C4—C5—C6	−177.0 (3)
C1—C2—C3—C4	−178.7 (3)	C4—C5—C6—C7	177.6 (3)
C2—C3—C4—C5	178.8 (3)	C5—C6—C7—N2	−65.4 (4)

### Hydrogen-bond geometry (Å, °)

**Table:**

*D*—H···*A*	*D*—H	H···*A*	*D*···*A*	*D*—H···*A*
N1—H11···I1	0.88 (2)	2.98 (4)	3.738 (4)	145 (5)
N1—H12···I1^i^	0.90 (2)	2.88 (3)	3.706 (3)	153 (3)
N2—H21···N1^ii^	0.90 (2)	1.87 (3)	2.740 (4)	164 (5)
N2—H22···I1^iii^	0.87 (2)	2.72 (2)	3.579 (3)	170 (3)
N2—H23···I1^iv^	0.90 (2)	2.83 (2)	3.646 (3)	152 (2)

Symmetry codes: (i) *x*, *y*, *z*+1; (ii) −*x*+2, *y*+1/2, −*z*+1; (iii) −*x*+1, *y*+1/2, −*z*; (iv) −*x*+1, *y*+1/2, −*z*+1.
